# Efficient and Rapid Microfluidics Production of Bio-Inspired Nanoparticles Derived from *Bombyx mori* Silkworm for Enhanced Breast Cancer Treatment

**DOI:** 10.3390/pharmaceutics17010095

**Published:** 2025-01-12

**Authors:** Muhamad Hawari Mansor, Zijian Gao, Faith Howard, Jordan MacInnes, Xiubo Zhao, Munitta Muthana

**Affiliations:** 1 School of Medicine and Population Health, The University of Sheffield, Barber House, Sheffield S10 2HQ, UK; m.h.mansor@sheffield.ac.uk (M.H.M.); zgao31@sheffield.ac.uk (Z.G.); f.howard@sheffield.ac.uk (F.H.); 2 School of Chemical, Materials and Biological Engineering, The University of Sheffield, Mappin Street, Sheffield S1 3JD, UK; j.m.macinnes@sheffield.ac.uk; 3 School of Pharmacy, Changzhou University, Changzhou 213164, China

**Keywords:** microfluidics, bio-inspired nanoparticles, curcumin, 5-fluorouracil, magnetic nanoparticles, breast cancer

## Abstract

**Background/Objectives**: In the quest for sustainable and biocompatible materials, silk fibroin (SF), derived from natural silk, has emerged as a promising candidate for nanoparticle production. This study aimed to fabricate silk fibroin particles (SFPs) using a novel swirl mixer previously presented by our group, evaluating their characteristics and suitability for drug delivery applications, including magnetic nanoparticles and dual-drug encapsulation with curcumin (CUR) and 5-fluorouracil (5-FU). **Methods**: SFPs were fabricated via microfluidics-assisted desolvation using a swirl mixer, ensuring precise mixing kinetics. Comprehensive physicochemical characterisation, including size, polydispersity index (PDI), zeta potential, and secondary structure analysis, was conducted. Further, CUR/5-FU-loaded magnetic core SFPs were assessed for cytotoxicity in vitro using breast cancer cell lines and for biodistribution and targeting efficiency in a murine breast cancer model. **Results**: The swirl mixer produced SFPs with sizes below 200 nm and uniform distributions (PDI < 0.20) with size stability for up to 30 days. Encapsulation efficiencies were 37% for CUR and 82% for 5-FU, with sustained drug release profiles showing 50% of CUR and 70% of 5-FU released over 72 h. In vitro studies demonstrated sustained cytotoxic effects, and cell cycle arrest at the G2/M phase in breast cancer cell lines, with minimal toxicity in non-cancerous cells. Cellular uptake assays confirmed efficient drug delivery to the cytoplasm. In vivo biodistribution studies revealed increased tumour-specific drug accumulation with magnetic guidance. Haematoxylin & Eosin (H&E) staining indicated enhanced tumour necrosis in treated groups compared to controls. **Conclusions**: This study underscores the utility of the swirl mixer for efficient and scalable fabrication of bio-inspired SFPs, supporting their application in targeted cancer drug delivery. These findings align with and advance previous insights into the use of microfluidics and desolvation methods, paving the way for improved therapeutic strategies in breast cancer treatment.

## 1. Introduction

In recent years, biomedical research has witnessed a surge in the exploration of bio-inspired materials for nanoparticle engineering. These materials are inspired by natural biomolecules such as proteins and lipids, offering improved tailoring of material properties, which makes them suitable for applications where interactions with living organisms are essential [1]. Notably, nanoparticles, which are commonly employed as drug carriers, benefit significantly from the utilisation of these bio-inspired materials. Furthermore, these materials are often produced using environmentally friendly processes found in nature, leading to greener and more sustainable methods of material synthesis and ultimately reducing the environmental impact of production [2]. At the forefront of this innovative approach is the utilisation of natural proteins, particularly silk fibroin (SF), which is derived from silk produced by the silkworm, *Bombyx mori*. SF possesses advantageous attributes for nanoparticle development, including biodegradability, non-antigenicity, and exceptional biocompatibility with the human body [3,4,5]. Therefore, this study sought to investigate the potential of silk fibroin particles (SFPs) formed using various techniques, guided by the principles of bio-inspired materials.

Despite the promise of protein-based nanoparticles such as SFPs, significant challenges persist in their production. These challenges are particularly pronounced when aiming for precise control over nanoparticle properties, making the scaled-up production inefficient. Protein-based nanoparticles are inherently sensitive to environmental factors such as temperature and pH, leading to variations and inconsistencies in their formation [6,7,8]. The achievement of uniform and reproducible outcomes has become a formidable task. Various methodologies have been documented, including desolvation [9,10,11], salting-out [12,13], supercritical fluid technology [14], and electric fields [15]. Among protein-based nanoparticles, SFPs produced through desolvation which involves the interplay between a solvent and solubilised or regenerated SF, have shown promise owing to their simplicity, feasibility, and cost-effectiveness. However, even with this method, challenges arise because of the substantial molecular weight of the SF protein [3].

To address these challenges, innovative techniques have emerged for nanoparticle formation with microfluidics leading the way. Microfluidics offers a highly efficient and precise method for the formation of nanoparticles. It is recognised for its ability to significantly reduce waste by precisely controlling chemical reactions and material formation at a microscale [16]. This level of control not only results in enhanced reproducibility and scalability, which are crucial aspects of nanoparticle production, but also aligns with environmentally friendly practices by minimising resource consumption. Various microfluidic devices have been developed to facilitate nanoparticle formation with varying results. These include the staggered herringbone mixer [17] and the T-junction mixer [18]. Each of these devices leverages the unique principles of fluid dynamics to assist nanoparticle formation.

However, we designed a microfluidic device based on a novel swirl mixer, which shows great potential. This instrument optimises the geometric design and mixing kinetics, enabling us to exert more precise control over the nanoparticle properties with high reproducibility. Specifically, it provides rapid and uniform mixing, addressing critical factors such as mixing time, growth time, and strain rate to achieve a consistent nanoparticle size and distribution [19,20,21]. Concurrently, we incorporated magnetic iron nanoparticles (MNPs) into SFPs. Magnetic guidance offers substantial potential for improving the efficacy and specificity of drug delivery systems. By harnessing the power of microfluidics, particularly through the implementation of a swirl mixer, we aim to significantly speed up the production of silk nanoparticles, improve drug encapsulation efficiency, and open up scaling opportunities while minimising material usage and wastage. This advancement will not only improve bio-inspired nanoparticle fabrication but also hold the promise of expanding their applications in biomedicine.

In this study, we conducted a comparative analysis of various production techniques, including salting out with phosphate buffer, standard batch desolvation, and microfluidics-assisted desolvation, using both a conventional T-mixer and an innovative swirl mixer. Our investigation explored the influence of diverse factors, such as solvent choice, total flow rate, silk-to-solvent ratio, and silk concentration on the physical attributes of the formed silk nanoparticles. These attributes include particle size, distribution, zeta potential, stability, secondary structure, and morphology. Additionally, we investigated the encapsulation of magnetic nanoparticles and two polar anticancer agents, curcumin and 5-fluorouracil (CUR/5-FU), using a swirl mixer approach. The resulting complexes underwent a series of chemical and physical characterisations, including Fourier-transform infrared spectroscopy (FT-IR), transmission electron microscopy (TEM), and vibrating sample magnetometry (VSM). Subsequently, we conducted tests using the formed nanoparticles on three distinct breast cancer cell lines, MCF-7, SK-BR-3, and MDA-MB-231, as well as an immortalised human embryonic kidney cell line, HEK-231, to assess cytotoxicity and cellular uptake. Finally, we performed in vivo tolerability and biodistribution studies to examine any potential adverse reactions associated with drug administration and evaluated the targeting capability of magnetic guidance using a mouse model of breast cancer.

## 2. Materials and Methods

### 2.1. Materials

*Bombyx mori* silk was obtained from Jiangsu, China. The following chemicals and reagents were used: Na_2_CO_3_ (11552), 5-fluorouracil (A13456), and curcumin (B21573) from Alfa Aesar, Haverhill, MA, USA; CaCl_2_ (C1016) from Sigma-Aldrich, St. Louis, MO, USA; Roswell Park Memorial Institute (RPMI) 1640 Medium with L-Glutamine (BE12-702F), Dulbecco’s Modified Eagle’s Medium (DMEM) with Ultraglutamine 1 with 4.5 g/L Glucose (BE12-604F/U1), 5000 U/mL penicillin-5000 U/mL streptomycin (DE17-603E), foetal bovine serum (FBS) (FB1001), and Dulbecco’s Phosphate Buffered Saline (DPBS) without Calcium or Magnesium (17-512F) from Lonza Group AG, Basel, Switzerland. The 3-(4,5-Dimethylthiazol-2-yl)-2,5-Diphenyltetrazolium Bromide (MTT) (M6494) assay, 4′,6-diamidino-2-phenylindole, dihydrochloride (DAPI) (D1306), Vybrant™ DiD Cell-Labeling Solution (V22887), PI/RNase Staining Solution (F10797), and Trypan Blue (15250) were purchased from Thermo Fisher Scientific, Waltham, MA, USA. Human adenocarcinoma cells (MCF-7 (HTB-22), MDA-MB-231 (HTB-26), and SK-BR-3 (HTB-30)), murine cell 4T1 (CRL-2539) and HEK293 cells (CRL-1573) were obtained from American Type Culture Collection (ATCC, Manassas, VA, USA).

### 2.2. Preparation of Different Silk Nanoparticles Formation Process

**Figure 1:**
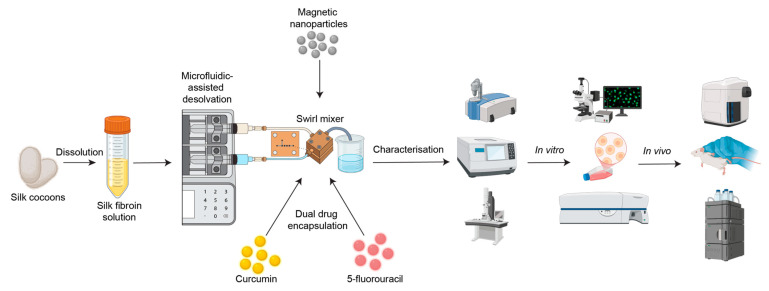
Schematic overview of the workflow for silk fibroin particle (SFP) production using microfluidics-assisted desolvation. Silk cocoons are dissolved to produce silk fibroin solution, followed by nanoparticle fabrication using a custom swirl mixer. The resulting particles are characterised using various methods, such as dynamic light scattering (DLS), Fourier-transform infrared spectroscopy (FTIR), and transmission electron microscopy (TEM). Dual drug encapsulation (curcumin and 5-fluorouracil) is assessed through in vitro studies (e.g., fluorescence microscopy and flow cytometry) and in vivo biodistribution studies (e.g., haematoxylin and eosin (H&E) staining and high-performance liquid chromatography (HPLC)).

Silk fibroin nanoparticles (SFPs) were fabricated using a microfluidics-assisted desolvation method with a swirl mixer designed for enhanced mixing efficiency and reproducibility. The schematic in Figure 1 illustrates the workflow from silk fibroin preparation to nanoparticle fabrication, drug encapsulation, and evaluation of therapeutic efficacy through in vitro and in vivo studies. The swirl mixer microfluidic devices used in this study were developed by our research group and featured swirl microchannels with four mixing elements designed to enhance fluid mixing efficiency and reproducibility. These devices have been previously employed in studies by Tomeh, Mansor [22] and Gao, Mansor [23], showcasing their reliability in nanoparticle formation.

The microfluidic chips were fabricated using laser machining on durable polymer sheets to create precise chamber geometries and channel structures. The chips were securely clamped between supporting blocks equipped with inlet and outlet connection points. The leak-proof operation was achieved through the incorporation of sealing mechanisms, ensuring consistent performance during fluid flow. The system was connected to a dual-syringe pump, which enabled precise regulation of flow rates. Reagents were introduced via inlet channels, and the swirl microchannels generated cyclonic motion, ensuring thorough mixing at the microscale. In the microfluidic approach for the swirl mixer, the silk solution and precipitating agent were introduced into the device through syringe inlets. The dynamic mixing achieved within the swirl microchannels facilitated rapid and uniform nanoparticle formation.

### 2.3. Silk Fibroin Solution Preparation

The production of silk fibroin (SF) aqueous stock solutions was produced as previously described by Haider et al. [24]. Briefly, approximately 5 g of silk fibroin (SF) was degummed by boiling in 2 L of 0.02 M sodium carbonate (Na_2_CO_3_) solution for 30–90 min, followed by thorough rinsing with hot distilled water (dH_2_O) and drying at 60 °C for 2 days. Degumming typically yielded ~3.5 g of SF, ensuring effective sericin removal.

The dried SF was dissolved in Ajisawa’s reagent (1:2:8 molar ratio of CaCl_2_: ethanol: deionised water) at 70 °C for 3 h under constant stirring. The resulting solution was dialysed using a membrane (MWCO: 12 kDa) in 2 L of dH_2_O for up to 3 days, with regular water replacement. Conductivity testing ensured a final conductivity of <10 µS.

The dialysed SF solution was centrifuged twice at 8500–9500× *g* for 20 min at 4–6 °C to remove impurities. The concentration of the silk solution (*w*/*v*) was determined by drying a known volume (200 µL) overnight at 60 °C and calculating the difference in weight. The final SF solution was stored at 4 °C in aliquots to minimise degradation.

### 2.4. Preparation of SFPs

Silk fibroin nanoparticles (SFPs) were fabricated using a microfluidics-assisted desolvation method with a custom-designed swirl mixer. The swirl mixer, developed as a prototype with unique mixing geometry, facilitates precise and rapid mixing of silk fibroin solutions with organic solvents, enabling controlled nanoparticle formation. The resulting nanoparticle suspensions were collected, stabilised in a dilution medium (e.g., dH_2_O), and purified using ultrafiltration membranes (Pall Macrosep^®^ 100 K). The SFPs were subsequently stored at 4 °C for further analysis. Detailed descriptions of the swirl mixer design, operation, and methodology have been reported previously and can be found in Tomeh, Mansor [22].

### 2.5. Particle Size and Zeta Potential Analysis

DLS analysis was performed using a NanoBrook 90 plus Pals Particle Size Analyser (Brookhaven Instrument, Nova Instruments, New York, NY, USA) to monitor changes in the hydrodynamic diameter (Dh) or size, PDI, and zeta potential of SFPs. These parameters are essential for assessing the stability and characterising the physical properties of SFP dispersions over time. To conduct measurements, the particles were suspended in DI water at an appropriate concentration and placed in a cuvette for size analysis. The measurements were performed at 25 °C using a He/Ne 660 nm laser. Each run consisted of five repetitions with a duration of 45 s per run. Refractive indices of 1.331 for water and 1.600 for particles were used to accurately determine the particle size. For zeta potential measurements, the pH of each sample was adjusted to 7 before analysis. Zeta potential values were determined using the same instrument. The size characterisation steps were followed, but the electrode was fitted into the cuvette prior to measurement. Each sample was measured in triplicates to ensure data accuracy and reliability.

### 2.6. Determination of SFPs Secondary Structure Percentage

The SFPs suspension was first frozen at −80 °C for 12 h to ensure complete freezing and subsequently subjected to freeze-drying using a ScanVac Coolsafe Freeze Dryer (LaboGene, Allerød, Denmark) at a vacuum pressure of 0.01 mbar and a condenser temperature of −50 °C for 24 h. The resulting freeze-dried SFPs were thoroughly mixed with ground potassium bromide (KBr) to ensure homogeneity and pressed into solid discs. Secondary structure analysis was performed using Fourier-transform infrared (FT-IR) spectroscopy (PerkinElmer Frontier FT-IR, Perkin Elmer, Shelton, CT, USA) over the wavenumber range 400–4000 cm^−1^. Each measurement consisted of 128 scans, with a resolution of 4 cm^−1^. To correct the baseline and conduct peak fitting, the amide I region (1575–1750 cm^−1^) was utilised based on previous studies [25]. Spectral data analysis was performed using default software provided by the IR machine.

### 2.7. Yield Quantification of Formed SFPs

To determine the percentage yield of SFPs, the volume of purified SFPs in the suspension obtained from the mixer outlet was calculated. Five empty Eppendorf tubes (1.5 mL) were weighed (*W*1). The SFP suspension (1 mL of SFP suspension was added to each tube and the tubes were then dried in an oven at 40 °C for 24 h. The total weight of the tubes containing SFPs was recorded (*W*2). The yield was calculated using the following Equation (1):

(1) $$\%\;Yield\;of\;the\;silk\;nanoparticles=\frac{\left(W2-W1\right)\times 1\;mL}{Initial\;concentration\;of\;added\;SF\times 1\;mL}$$

### 2.8. Stability Analysis of SFPs in PBS

The formed SFPs were stored in PBS at 4 °C to evaluate their stability. The size, PDI, and zeta potential of the SFPs were determined using DLS at specific time points: days 0, 10, 20, and 30. To ensure data reliability and reproducibility, each sample was analysed in at least three independent replicates.

### 2.9. Fabrication of Magnetic Nanoparticles Using Swirl Mixer

Iron oxide (Fe_3_O_4_) nanoparticles were synthesised using a modified co-precipitation method, as described by Besenhard, LaGrow [26], adapted for use with the swirl mixer. In this method, precursor solutions of iron salts were prepared and combined under an inert atmosphere to prevent oxidation. These solutions, along with a base solution, were introduced into the swirl mixer, where controlled and efficient mixing facilitated the precipitation of Fe_3_O_4_ nanoparticles. The resulting nanoparticle suspension was subsequently neutralised with acid, purified through multiple washing steps, and either dried or suspended in deionised water for storage. This modified approach leverages the swirl mixer’s design to achieve enhanced mixing efficiency and uniform nanoparticle formation.

### 2.10. Preparation of Magnetic Core-Shell Nanoparticles Using the Novel Swirl Mixer

Magnetic core silk nanoparticles (MSFPs) were fabricated to evaluate the swirl mixer performance. Initially, a solution of magnetic nanoparticles (<50 nm) was diluted to a concentration of 1.0 mg/mL in the silk solution. The prepared solution was loaded into a syringe connected to a syringe pump. The formation of MSFPs followed a procedure similar to that of SFPs. After fabrication, the resulting MSFPs were collected using a strong neodymium magnet, rinsed in distilled water to remove any residual material, and finally resuspended in PBS prior to storage.

### 2.11. Encapsulation and Release Efficiency of Curcumin and 5-Fluorouracil

To prepare the stock solutions of curcumin (CUR) and 5-Fluorouracil (5-FU), a concentration of 1 mg/mL was achieved by dissolving the drugs in dimethyl sulfoxide (DMSO) and DI water, respectively. The volume of each solution was adjusted accordingly and then diluted in acetone for CUR and in silk solution for 5-FU in preparation for the fabrication of drug-loaded magnetic core silk nanoparticles (CF-MSFPs). Following the CF-MSFP formation, the resulting particles were collected using a magnet and subsequently washed multiple times with DI water to remove any residual material. To quantify the amount of encapsulated drug, the supernatant containing free drug was collected and measured at 424 nm (CUR) and 266 nm (5-FU) using a UV-Vis spectrophotometer (JENWAY 6715, Bibby Scientific, Stafford, UK). Standard curves for CUR and 5-FU were established under the same measurement conditions. The linear correlation between different drug concentrations and UV absorbance was determined within a concentration range of 0–200 µg/mL (R2 = 0.99). Encapsulation efficiency (*EE*%) was calculated using the following Equation (2):

(2) $$EE\%=\left(\frac{\left(M_{0}-M_{1}\right)}{M_{0}}\right)\times 100\%,$$

where *M*_1_ represents the mass of the free drug (mg) and *M*_0_ is the initial mass of the drug added to the solution (mg). To evaluate the release percentage of the drugs from the particles, suspensions of CUR/5-FU-loaded MSFPs were diluted to a concentration of approximately 1 mg/mL. The diluted suspensions were then placed in a dialysis membrane with a molecular weight cutoff of 12 kDa and immersed in 1 L of PBS. The total drug concentration released from the dialysis tube at different time points was measured by sampling a specific volume of PBS buffer and measuring it using a UV-Vis spectrophotometer. The release percentage was calculated using the equation mentioned above, where *M*_0_ represents the initial mass of the drug in the particles (mg), and *M*_1_ is the mass of the drug released (mg).

### 2.12. Fourier-Transformed Infrared Spectroscopy Analysis

The chemical composition and functional groups of the designed nanoparticles were investigated using an FT-IR machine (IR Prestige-21, Shimadzu, Kyoto, Japan). FT-IR spectra were obtained over a wavenumber range of 400–4000 cm^−1^. Data acquisition involved 64 scans with a resolution of 4 cm−1 and was processed using Happ–Genzel apodization.

### 2.13. Transmission Electron Microscopy Imaging of SFPs

Transmission electron microscopy (TEM) was utilised to capture electron images of the formed SFPs, MNPs, MSFPs, and CF-MSFPs using an FEI Tecnai G2 Spirit BioTWIN microscope (Field Electron and Ion Company, Hillsboro, OR, USA) operating at an accelerating voltage of 80 kV. The samples were placed on TEM carbon grids and air-dried prior to analysis. For the visualisation of SFPs, the grids with the samples were dried and subsequently stained with 0.1% (*w*/*v*) phosphotungstic acid for 1 min. The resulting TEM images were captured using a dedicated camera positioned underneath the TEM stage and further processed for analysis.

### 2.14. Fluorescence Microscopy Imaging of Drug Loaded MSFPs Cellular Uptake

Qualitative analysis of the cellular uptake of drug-loaded magnetic core silk nanoparticles (MSFPs) in MDA-MB231 cells was performed using a Zeiss LSM 980 fluorescence microscope (Carl Zeiss Ag, Oberkochen, Germany). The cancer cells were initially cultured for 24 h at 37 °C and then treated with the particles. After co-incubation for an additional 24 h, the cells were rinsed with PBS, fixed with 3% paraformaldehyde for 20 min at room temperature, and stained with DAPI (0.1 µg/mL) and DID (5 µM) for 10 min and 15 min, respectively. Subsequently, fluorescence microscopy was employed to image the cells and analyse the cellular uptake of the MSFPs. Imaging conditions included excitation wavelengths of 405 nm for DAPI and 644 nm for DiD. Images were captured with identical exposure settings for all samples to ensure comparability. Each experiment was repeated three times to ensure reproducibility and accuracy.

### 2.15. In Vitro Cytotoxicity Assay

In this study, adenocarcinoma breast cancer cells, specifically MDA-MB-231, MCF-7, and SK-BR-3, were used to investigate the cytotoxic effects of SFPs. Additionally, the non-cancerous immortalised cell line HEK-293 was included in the analysis. MDA-MB-231, MCF-7, and HEK-293 cells were cultured in DMEM medium, while SK-BR-3 cells were grown in RPMI medium, all maintained at 37 °C with 5% CO_2_. The cell culture medium was supplemented with 10% FBS, 1% penicillin/streptomycin, and 1% fungizone to promote cell growth and viability. The cytotoxicity assay was performed using the MTT assay. Briefly, 1 × 10^4^ cells were seeded into each well of 96-well plates and allowed to incubate for 24 h to facilitate attachment. Subsequently, the cells were exposed to all SFP formulations as well as free CUR and 5-FU at selected concentrations for 2 days. After the incubation period, the old medium was removed, and a mixture of MTT and fresh medium was added to the cells to assess cell viability. Further analyses were conducted to evaluate the cytotoxic effects of the different formulations on breast cancer and non-cancerous cell lines.

### 2.16. In Vivo Tolerability Study

In the in vivo study, female BALB/c mice were implanted with murine breast cancer cells (4T1 cells in a 1:1 mixture of PBS:GFR-Matrigel) in the mammary fat pad. The mice were monitored until the tumours grew to a size of approximately 100 mm^3^. Subsequently, the treatment phase was initiated and involved three different treatment groups: PBS, CF-MSFP, and free CUR and 5-FU (CF). The dosages of the drugs were 25 mg/kg for CUR and 15 mg/kg for 5-FU, and the same dosages were used for the drugs encapsulated in SFPs. In the CF-MSFP treatment group, the right tumour was subjected to a magnetic gradient of 1 cm depth using three neodymium magnets for 30 min after each treatment session. All treatments were administered every 3 days for a total of three treatment sessions. Throughout the study, the body weight of the mice and the tumour size were measured daily after the initial implantation. After the last treatment, all mice were euthanised 24 h later, and their organs were harvested for further analysis. This experimental design aimed to investigate the effects of CF-MSFPs in comparison to free CF on tumour growth and potential organ-specific drug distribution. The magnetic targeting approach is utilised to enhance drug delivery to the tumour site and assess its impact on treatment efficacy.

**Figure 2:**
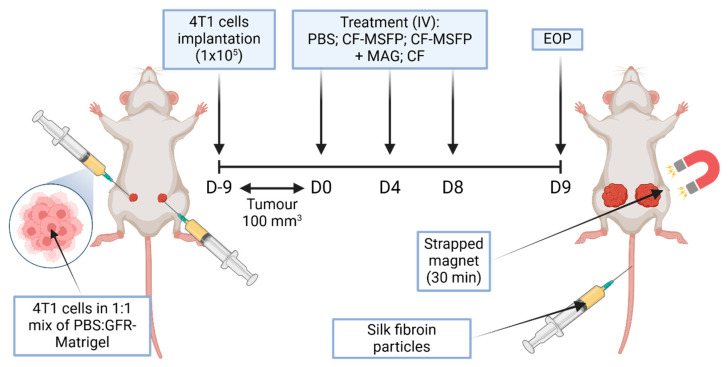
Schematic overview of in vivo study. This figure represents a schematic overview of a study investigating the efficacy of magnetically-guided silk fibroin particles with encapsulated curcumin and 5-fluorouracil (CF-MSFP) for targeted breast cancer therapy. In the depicted model, 4T1 breast cancer cells were implanted bilaterally into a BALB/c mouse. Following tumour establishment, the mouse received intravenous treatments of CF-MSFP with and without magnetic guidance (MAG) applied to the right tumour, and free drug (CF) as a control. The magnet was strapped in place for 30 min post-treatment to localize the magnetic nanoparticles to the tumour site, aiming to enhance therapeutic concentration and minimize systemic side effects.

This experimental setup was designed to compare the targeted delivery capability and therapeutic efficacy of SFPs loaded with chemotherapeutic agents, CUR, and 5-FU in a murine model of breast cancer (Figure 2). Magnetic guidance was employed to potentially enhance particle accumulation in the tumour tissue. The bilateral tumour implantation into the mammary fat pad allows for a within-subject comparison, where each mouse serves as its own control, ensuring a more accurate assessment of the magnetic targeting system’s impact on drug delivery and tumour response. This study aims to advance the precision of cancer therapies by leveraging the properties of magnetic nanoparticles for improved drug localization and tumour treatment outcomes.

### 2.17. Necrosis Quantification Assay

In this study, the tumour tissue harvested from the mice was processed for further analysis. First, the tumour tissue samples were carefully frozen in Optimal Cutting Temperature (OCT) compound to preserve their integrity. Subsequently, the frozen tumour tissue blocks were sectioned into 10-micron thick slices using a cryostat machine (CryoStar™ NX70, Thermo Fisher Scientific, Waltham, MA, USA). These sections were then subjected to Haematoxylin and Eosin (H&E) staining using standard procedures to provide a clear visualisation of the tissue morphology, making it easier to identify necrotic regions within the tumour tissue. High-resolution images of the stained tumour tissue slides were captured using a slide scanner to ensure an accurate and detailed representation of the tissue structure. The captured images were then analysed using the QuPath 0.50 (QuPath Image Analysis Software, University of Edinburgh, Edinburgh, UK) to determine the percentage of necrosis present in the tumour tissue.

### 2.18. Curcumin and 5-Fluorouracil Biodistribution Study

For biodistribution analysis, all tissues obtained from the mice were homogenised to remove cells, and the resulting supernatant was collected. This supernatant was then analysed using HPLC. The analysis was conducted using a C18 reverse-phase column (150 mm × 4.6 mm, 5 μm particle size) with a mobile phase of acetonitrile:water (70:30, *v*/*v*) at a flow rate of 1.0 mL/min. The detection wavelength was set at 254 nm, and the column temperature was maintained at 30 °C to ensure optimal separation. Before analysis, standard curves for both CUR and 5-FU were generated. The standard curve served as a reference to quantify the drug concentrations in the collected tissue samples. Tissue homogenates were subjected to HPLC analysis, and the concentrations of CUR and 5-FU in each tissue sample were determined by comparing the results with the respective standard curves. By conducting this biodistribution analysis, we aimed to investigate how CF-MSFPs are distributed among different tissues in mice, with and without magnetic targeting.

### 2.19. Statistical Analysis

All statistical analyses were performed using GraphPad Prism 10 software (GraphPad Inc., San Diego, CA, USA). Quantification of immunohistochemistry (IHC) staining was carried out using QuPath 0.50 (University of Edinburgh, Edinburgh, UK). Normality tests were conducted for each dataset using the D’Agostino–Pearson test or Shapiro–Wilk test within Prism to ensure the data followed a normal distribution. Then, the student’s *t*-test was used to compare two groups, whereas one-way ANOVA and two-way ANOVA were used to compare multiple groups. All data are presented as the mean and standard deviation (SD), with a minimum of *n* = 3, unless specified otherwise in the figure legend. *p* values are as follows: * = *p* ≤ 0.05, ** = *p* ≤ 0.01, *** = *p* ≤ 0.001, and **** = *p* ≤ 0.0001.

## 3. Results

### 3.1. Size, PDI, and Zeta Potential of Silk Fibroin Particles (SFPs)

The silk fibroin particles (SFPs) fabricated using the swirl mixer exhibited an average particle size of approximately 150–200 nm, with a low polydispersity index (PDI) below 0.2. These findings indicate a uniform size distribution, which is critical for applications in drug delivery. Notably, the size and size distribution of the silk nanoparticles produced in this study aligned with those reported in previous studies by Perteghella, Crivelli [27], Seib, Jones [28], Sharma, Bano [29], Zhang, Shen [11], and Shaidani, Jacobus [30]. These authors demonstrated that the use of solvent led to the production of silk nanoparticles with sizes ranging from approximately 100 to 200 nm.

Zeta potential analysis revealed a surface charge of approximately −39 mV, signifying strong colloidal stability. The negative surface charge is attributed to the amino acid composition of silk fibroin, specifically the presence of aspartic and glutamic acids, which contribute to the formation of a negatively charged electric double layer [31]. This stabilising effect minimises aggregation and enhances the potential for long-term storage. Similar negatively charged zeta potential trends have been observed in silk fibroin nanoparticles fabricated through comparable desolvation processes [22,32,33,34].

### 3.2. Secondary Structure of SFPs

Fourier-transform infrared spectroscopy (FTIR) analysis of the amide I region was used to determine the secondary structure of the SFPs. The spectra revealed a significant increase in β-sheet content compared to unprocessed silk fibroin. The transformation into β-sheet structures during nanoparticle formation enhances mechanical robustness and contributes to the stability of the nanoparticles, making them suitable for biomedical applications such as drug delivery. This finding is consistent with previous research, which demonstrated similar increases in β-sheet content during silk fibroin nanoparticle fabrication via microfluidic and desolvation methods [5,35,36]. A higher percentage of β-sheet structure typically correlates with increased stability, making SFPs more suitable for long-term drug delivery applications [37].

### 3.3. Yield of SFPs

The swirl mixer achieved a yield of approximately 33% for SFPs, representing a significant improvement over the staggered herringbone micromixer (Nanoassemblr™), which reported yields of only 2–3% [17]. This highlights the efficiency of the swirl mixer in converting silk fibroin into nanoparticles with minimal material wastage.

While traditional salting-out methods can produce larger batches of SFPs, they are inherently time-intensive, requiring hours to produce the same volume achievable in less than 30 s with the swirl mixer. Additionally, scaling up the salting-out method poses significant challenges, particularly in maintaining consistency and optimising particle characteristics at high volumes. In contrast, the swirl mixer offers precise control over particle size and distribution, making it a more efficient and scalable option for nanoparticle production. This comparison underscores the advantages of the swirl mixer in high-throughput and industrial-scale applications, where consistency and efficiency are paramount.

### 3.4. Stability of SFPs in PBS

**Figure A1:**
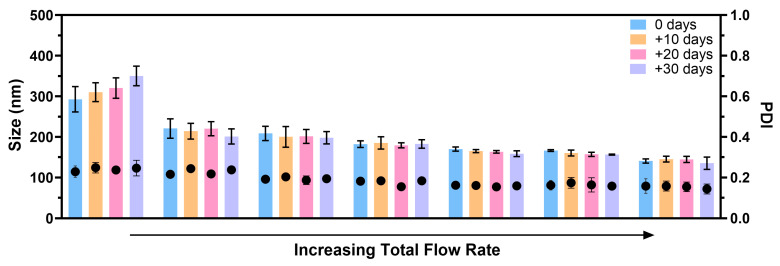
SFPs produced using the swirl mixer at different total flow rate demonstrated stability in size and PDI values for up to 30 days of storage at 4 °C. The stability of the silk nanoparticles was evaluated immediately after fabrication (day 0) and after 10, 20, and 30 days of storage. To ensure purity, the SFPs were subjected to dialysis immediately following fabrication. The results showed consistent particle size and distribution over the storage period, highlighting the effectiveness of the swirl mixer in producing stable silk fibroin nanoparticles. Error bars are hidden when not visible; data are presented as mean ± SD, *n* ≥ 3.

In this study, we explored the stability of fabricated SFPs in PBS at 4 °C. To assess stability, the particles were purified using dialysis to remove most of the solvents from the silk suspension. Subsequently, the SFPs were characterised using DLS on the day of formation and at three additional time points, each ten days apart from the formation day. The experiments were conducted using different total flow rates, ranging from lower to higher while maintaining a constant SF concentration and a silk-to-solvent ratio. The results obtained from Figure A1 in Appendix A reveal a noteworthy trend, where the stability of the SFPs increased with higher total flow rates. This observation can be attributed to the presence of larger particles in the suspensions fabricated at lower flow rates. Typically, larger particles are more prone to instability and tend to undergo structural changes, leading to the potential aggregation of smaller particles in the suspension. This phenomenon can be attributed to Ostwald ripening, where smaller particles, having higher solubility due to their greater surface energy, dissolve and redeposit material onto larger particles [38,39]. This process leads to an increase in particle size over time and broadening of the particle size distribution, resulting in aggregation. Additionally, DLS is highly sensitive to the presence of larger particles, and any variations in their presence can significantly impact the measurements and lead to inaccuracies.

In contrast, SFPs produced at higher flow rates demonstrated enhanced stability at 4 °C, with no significant size differences observed for up to 30 days. The consistent size and distribution of these nanoparticles over time point towards a more stable formulation, which is of crucial importance for potential biomedical applications such as drug delivery systems. The improved stability of SFPs at higher flow rates further highlights the significance of precise control over the formation parameters to optimise the particle characteristics for specific applications.

### 3.5. Loading and Release of Curcumin and 5-Fluorouracil

**Figure 3:**
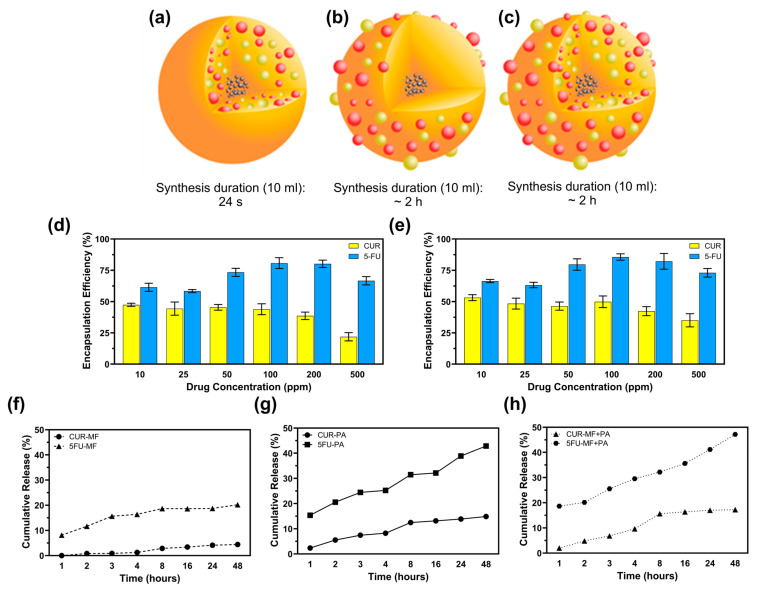
Microfluidic-assisted desolvation (MF) enables faster drug encapsulation at an efficiency comparable to that of conventional surface physical adsorption (PA). (**a**) CF-MSFPs were fabricated using a swirl mixer with a fabrication duration of 24 s. (**b**) CF-MSFPs were fabricated using a swirl mixer with a fabrication duration of approximately 2 h. (**c**) CF-MSFPs were fabricated using a swirl mixer with a fabrication duration of approximately 2 h, displaying a different encapsulation structure. (**d**) Encapsulation efficiency (EE) of CUR and 5-FU encapsulation in MSFPs fabricated using a swirl mixer. (**e**) Comparison of encapsulation efficiency at varying drug concentrations for CUR and 5-FU. (**f**) Cumulative release of CUR and 5-FU from MF-based encapsulation in PBS after 48 h. (**g**) Cumulative release profile of CUR and 5-FU using PA-based encapsulation. (**h**) Cumulative release of CUR and 5-FU with a combined MF + PA approach. Measurements were taken at regular intervals to determine encapsulation efficiency and release profile. Error bars are hidden when not visible, ±SD, *n* ≥ 3.

The encapsulation efficiency (EE) of curcumin (CUR) and 5-fluorouracil (5-FU) in magnetic MSFPs was investigated at six different drug concentrations using a microfluidic approach. The microfluidic device allowed for precise control over the mixing process, leading to the rapid formation and encapsulation of the drugs within the MSFPs. At a drug concentration of 200 ppm, approximately 37% of CUR and 82% of 5-FU were successfully encapsulated within MSFPs (Figure 3d,e). Comparing the microfluidics-based approach to conventional methods that rely on surface adsorption for attaching hydrophobic drugs to nanoparticles [40], it was observed that surface adsorption techniques often achieve higher encapsulation efficiency. However, these conventional methods may exhibit drawbacks in terms of drug release kinetics and protection of the loaded drugs [41]. During surface adsorption, drugs attach to the nanoparticle surface through electrostatic interactions, which can result in a higher initial encapsulation efficiency [19,42]. However, the release of drugs from nanoparticles tends to be faster owing to weak interactions, resulting in burst release effects [43,44,45]. Moreover, the loaded drugs may be less protected during storage and transit, thereby increasing the risk of drug degradation or premature release before reaching the target site.

In contrast, the microfluidics-based encapsulation approach used in this study offers several advantages. By leveraging the rapid and efficient mixing capabilities of the microfluidic device, the encapsulation process was completed in less than 20 s, minimising drug exposure to harsh conditions and potential degradation (Figure 3a–c). Additionally, the controlled release behaviour observed with the microfluidics-based MSFPs is particularly advantageous for cancer therapy. The sustained release of hydrophobic drugs, such as CUR, from MSFPs, allows for gradual and prolonged drug release, optimising treatment efficacy while reducing off-target effects.

Furthermore, the drug release profiles from the drug-loaded MSFPs (CF-MSFPs) indicated distinct release trends for CUR and 5-FU, with 5-FU dispersing more rapidly in PBS owing to its hydrophilic nature. However, the hydrophobic nature of CUR led to slower and sustained release from the MSFPs. Therefore, this controlled release behaviour is particularly valuable for cancer therapy, where a sequential and sustained release of drugs with varying properties can optimise treatment efficacy and minimise side effects.

Overall, the microfluidics-based encapsulation approach demonstrated in this study offers significant advantages over conventional methods by providing rapid and efficient drug loading into MSFPs. This technique opens new possibilities for developing precise and tailored drug delivery systems with enhanced control over drug release kinetics, paving the way for advanced and personalised cancer therapy.

### 3.6. Magnetic Moment Analysis

The presence of magnetic nanoparticles (MNPs) within the silk fibroin matrix was evaluated to confirm their structural and functional properties. While direct elemental analysis of the nanoparticles was not performed due to logistical constraints, the incorporation of magnetic nanoparticles based on iron oxides (Fe_3_O_4_) is well-documented in the literature for similar synthesis approaches. Encapsulation of MNPs within the silk fibroin (SF) matrix prevents the release of free iron ions, thereby mitigating the risk of Fe-induced oxidative stress or cell damage [46]. This encapsulation strategy enhances the biocompatibility and safety of the nanoparticles for therapeutic applications.

**Figure 4:**
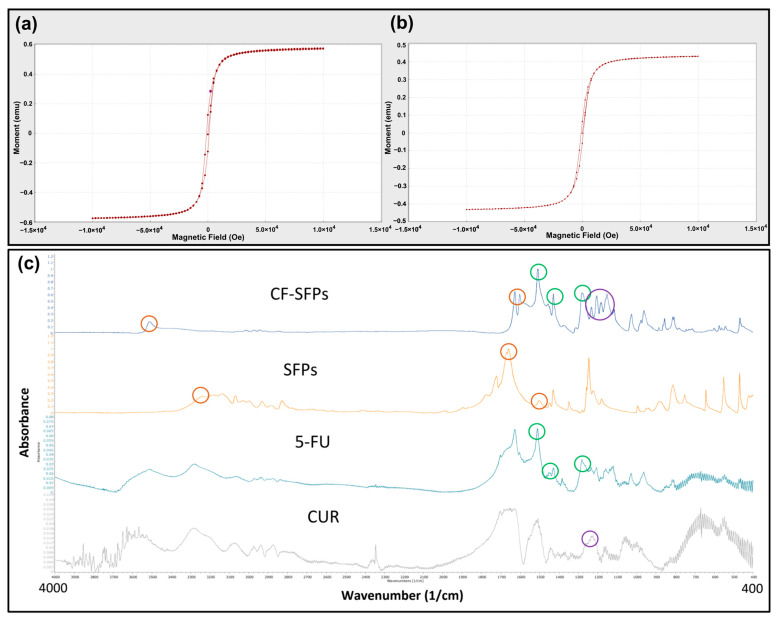
Microfluidics enabled the encapsulation of magnetic nanoparticles without excessive loss of magnetic strength and the successful incorporation of curcumin and 5-fluorouracil. VSM Analysis of MNPs (**a**) and MSFPs (**b**). The magnetic properties of the two different particle samples were compared. The formed samples were centrifuged to remove solvent and free MNPs, aliquoted into 1.5 mL centrifuge tubes, and placed in a carbon fibre tube holder at room temperature. Measurements were taken with a magnetic field ranging from −10,000 to 10,000 Oe. Figure (**a**) represents the MNPs in their native form, while figure (**b**) represents the MSFPs. A minor decrease in magnetic moment was observed when MNPs were encapsulated in SFPs. Graphical results obtained directly from the instrument. (**c**) FT-IR spectra of the designed CF-SFPs. FT-IR spectra for CUR, 5-FU, SFPs, and CF-SFPs. The coloured circles on each spectrum highlight characteristic functional groups pertinent to each substance: red circles for SFP, green circles for 5-FU, and purple circles for CUR. Optimised parameters were used for particle fabrication and 100 µg of CUR and 5-FU were added during the process. The presence of these groups in the final CF-SFPs spectrum, indicated by corresponding coloured circles, confirms the successful incorporation of CUR and 5-FU into the SFP matrix.

The magnetic properties of MSFPs were assessed using Vibrating Sample Magnetometry (VSM) (Figure 4a,b). The results showed that the MSFPs retained a similar magnetic moment to the native MNPs, albeit with a slight reduction. This reduction can be attributed to the presence of the SF coating surrounding the encapsulated MNPs. The SF coating acts as a protective layer, shielding the MNPs from direct exposure to the external environment, which may influence their magnetic properties. Additionally, the SF coating may introduce a slight hindrance to the mobility of the MNPs, leading to a marginal decrease in the overall magnetic moment.

These findings align with previous studies reporting similar observations. For instance, Del Bianco, Spizzo [47] demonstrated that embedding iron oxide superparamagnetic nanoparticles within a silk fibroin matrix reduced the magnetic moment due to the protective effect of the SF coating. Similarly, Greco, Schmuck [48] showed that artificial silk fibres containing magnetite nanoparticles exhibited a slight decrease in magnetic properties, attributed to the encapsulating silk fibroin matrix.

Overall, VSM analysis confirmed that the MSFPs retained their magnetic properties despite the encapsulation, making _them_ suitable candidates for magnetic-guided targeted drug delivery applications.

### 3.7. FTIR Analysis

For FTIR analysis, dual drug encapsulated forms of CF-SFPs without MNPs core were used. The presence of MNPs inside the complex does not need to be confirmed by FTIR as a magnet can be used to purify the sample. The results presented in Figure 4c show that SFPs showed characteristic peaks at 3309 cm^−1^, 1653 cm^−1^, and 1506 cm^−1^, which can be assigned to N–H stretching vibrations from amide groups, C=O stretching from the SF amide I structure, and N–H in-plane bending from the SF amide II structure, respectively. These peaks indicate the presence of SF as the main constituent of the nanoparticles. A small new peak at 1216 cm^−1^ emerged after CUR encapsulation, corresponding to the in-plane bending of aromatic CCH, which confirmed the successful incorporation of CUR within the SF nanoparticles. This observation is consistent with prior research demonstrating the integration of curcumin into silk fibroin matrices [49]. In addition to CUR, the FTIR spectra of CF-SFPs displayed additional characteristic peaks associated with 5-FU (5-FU). Specifically, the CF-MSFPs exhibited a new peak at 1594 cm^−1^, attributed to the C=O stretching vibration of 5-FU. Moreover, it also showed two distinct peaks at 1494 cm^−1^ and 1312 cm^−1^, corresponding to the N–H in-plane bending and C=O stretching vibrations of 5-FU, respectively. These spectral features confirm the successful encapsulation of 5-FU within the silk fibroin nanoparticles, as reported in similar studies [50]. The FTIR spectra confirmed the successful encapsulation of both CUR and 5-FU within the SF nanoparticles. Notably, shifts in peak positions and intensities suggest interactions between the drug molecules and the SF matrix, which may include hydrogen bonding or van der Waals forces, potentially indicating the formation of a stable drug-particle complex. The consistency of peak signatures across the spectra demonstrates the maintenance of the drugs’ molecular integrity within the composite structure.

Additionally, the FTIR spectra also show a broadening and disappearance of the sharp characteristic peaks typically observed for crystalline CUR and 5-FU in their pure forms. In the drug-loaded CF-SFPs, the absence of these sharp peaks, particularly in the regions around 1650 cm^−1^ and 1100 cm^−1^, indicates that both CUR and 5-FU were successfully encapsulated in an amorphous state [51]. This amorphous form is advantageous as it can enhance drug solubility, stability, and controlled release properties [52].

### 3.8. Transmission Electron Microscopy Imaging of SFPs

**Figure 5:**
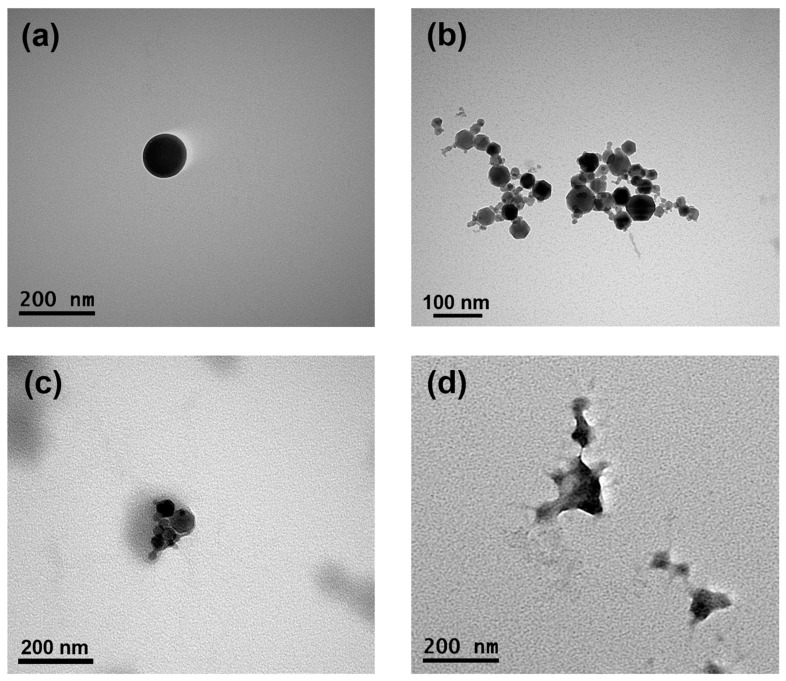
The encapsulation of magnetic nanoparticles and drugs changes the morphology of SFPs while retaining their size. Representative transmission electron microscopy images of SFPs (**a**), MNPs (**b**), MSFPs (**c**), and CF-MSFPs (**d**) produced using microfluidic-assisted desolvation in a swirl mixer. Optimised parameters were used for particle fabrication and 100 µg of CUR and 5-FU were added during the process. The scale bar indicates each image.

The morphological properties of the formed SFPs, MNPs, MSFPs, and CUR/5-FU-loaded MSFPs were visualised using TEM to gain insights into their structural characteristics (Figure 5). TEM images of the SFPs revealed the presence of round-shaped particles with sizes of approximately 120 nm, slightly smaller than the sizes reported in previous studies using DLS. While TEM provides high-resolution images of individual particles, DLS measures the hydrodynamic diameter of particles in solution, often resulting in a larger apparent size owing to the inclusion of solvation layers. The globular and dense particle shape of the SFPs (Figure 5a) can be attributed to the β-sheet structure, as reported in earlier studies [47]. On the other hand, the MNPs (Figure 5b) exhibited clusters of symmetrical hexagon-shaped particles, each measuring less than 50 nm in size. These results were consistent with the data obtained from the DLS analysis. Upon the addition of MNPs and drugs to the SFPs, the morphology of the particles underwent significant changes (Figure 5c,d). The TEM images of the MSFPs indicated a slightly distorted structure, with a dense core of MNPs surrounded by a lighter coating of SF. This observation supports the successful encapsulation of MNPs within SFPs to form MSFPs.

Furthermore, with the incorporation of CUR and 5-FU into the MSFPs, the spherical properties of the particles were altered, but distinct electron density differences were retained. This observation suggested that the core, primarily composed of MNPs and drugs, exhibited a higher electron density than the surrounding SF coating. The TEM results provided valuable insights into the morphology and structural arrangement of the formed nanoparticles. The successful encapsulation of MNPs and drugs within SFPs, as demonstrated by TEM imaging, validates the efficacy of the microfluidics-based approach for drug delivery applications. Moreover, understanding the structural characteristics of CUR/5-FU-loaded MSFPs aids in optimising their behaviour for targeted drug delivery and controlled release at the tumour site. Overall, TEM analysis complements the characterisation data obtained from other techniques, contributing to a comprehensive understanding of the physical properties of the formed nanoparticles.

### 3.9. CF-MSFPs Cellular Uptake Analysis

**Figure 6:**
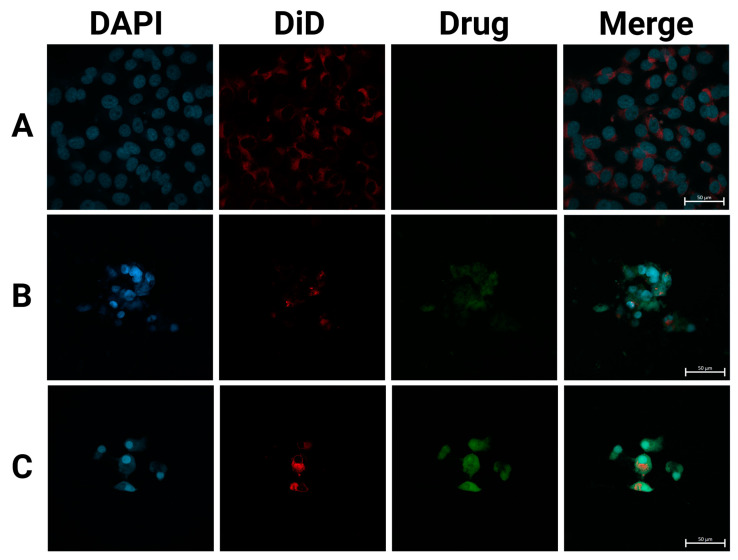
SFPs increased the cellular uptake of CUR/5-FU. Fluorescence images of MDA-MB-231 cells treated with PBS (**A**), CUR (**B**), and CF-MSFPs (**C**) for 24 h. Optimised parameters were used for particle fabrication and 100 µg of CUR and 5-FU were added during the process. The cell nucleus and cytoskeleton were stained by DAPI (blue) and DiD (red), respectively. The CUR is auto-fluorescent and can be detected using a fluorescent microscope. These data demonstrates the successful internalisation of CF-MSFPs into the cells, but it is not intended to quantify the drug release. Further studies are required to analyse the release kinetics and therapeutic efficacy of the encapsulated drugs.

Next, we performed fluorescence microscopy of the three different treatments following uptake by breast cancer cells. This included a negative control (cells with PBS), free CUR and 5-FU, and the particles containing CUR and 5-FU. The imaging results (Figure 6) revealed distinct patterns of drug localisation within the cells. The free drugs, CUR and 5-FU, appeared to be mostly distributed outside of the cell boundaries, indicating limited cellular uptake. This observation is consistent with the challenges faced by free drugs in crossing the hydrophobic cellular membrane and efficiently entering the cytoplasm.

In contrast, CF-MSFPs displayed a prominent presence of drugs inside the cytoplasm of the cells. The particles act as drug carriers, effectively delivering therapeutic agents to the target site. Despite the high negative zeta potential of silk fibroin nanoparticles and the negatively charged cell membrane, multiple mechanisms enable their effective cellular penetration. First, the nanoparticles’ size (~200 nm) is optimal for clathrin-mediated endocytosis, a pathway that allows efficient cellular uptake by overcoming the electrostatic repulsion barrier. Additionally, for magnetic silk fibroin particles (MSFPs), the application of an external magnetic field enhances delivery efficiency by directing the particles toward the cell surface, increasing their local concentration and facilitating uptake. Receptor-mediated endocytosis may further contribute to nanoparticle internalisation, as specific functional groups on the silk fibroin surface can interact with cell membrane receptors, enabling charge-independent cellular entry [53].

Once internalised, the nanoparticles release their cargo of CUR and 5-FU, allowing the drugs to exert their cytotoxic effects on cancer cells directly. These findings are consistent with previous studies that have demonstrated the superior cellular uptake of nanoparticle-based drug delivery systems compared to free drugs. For instance, research has shown that nanoparticles can enhance the intracellular delivery of therapeutic agents, overcoming the limitations associated with free drug administration [54].

### 3.10. In Vitro Cytotoxicity Assay

**Figure 7:**
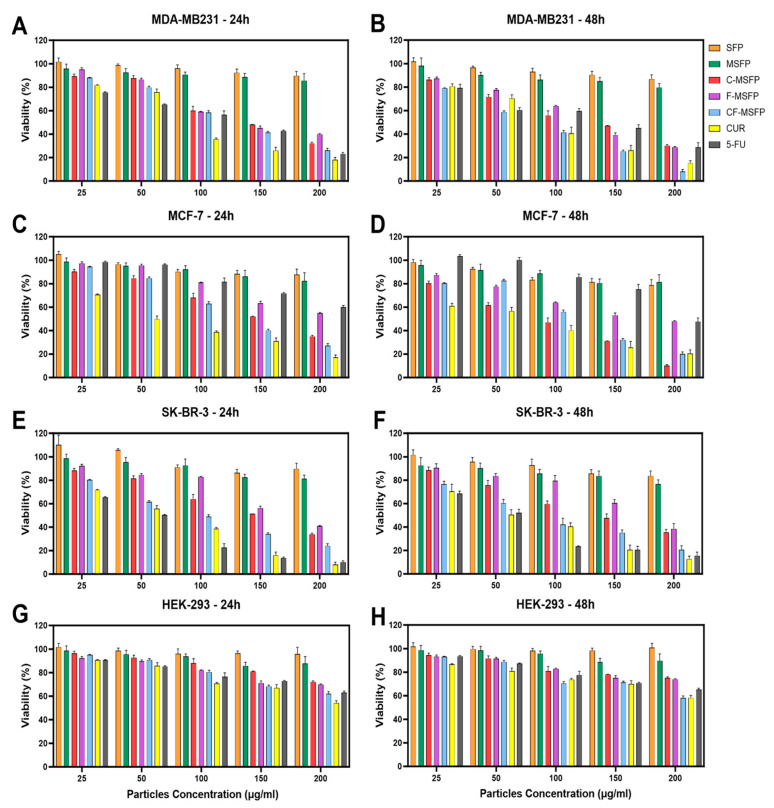
Cytotoxicity study of SFPs in different breast cancer cell lines after 24 and 48 h of treatment. The cancer cell viability after 24 and 48 h of treatment with different particle concentrations was determined using the MTT assay. SFP refers to silk fibroin particles, MSFP to magnetic core silk fibroin particles, C-MSFP to curcumin-encapsulated MSFP, F-MSFP to 5-Fluorouracil-encapsulated MSFP, CF-MSFP to curcumin and 5-Fluorouracil-encapsulated MSFP, CUR to free curcumin, and 5-FU to free 5-Fluorouracil. (**A**,**C**,**E**) show the toxicity of the different particle complexes and free drugs on MDA-MB231, MCF-7, and SK-BR-3 cells after 24 h of treatment, respectively. (**B**,**D**,**F**) show the same cells after 48 h of treatment. In contrast, figures (**G**,**H**) show the viability of HEK-293 cells (non-cancerous immortalised cell line) after 24 and 48 h of treatment, respectively. Data normalised to empty media control and statistical analysis was conducted using two-way ANOVA followed by post-hoc Tukey’s multiple comparison test. The detailed statistical analysis is not shown in the graph for clarity. Only four key comparisons (SFP vs. CF-MSFP, C-MSFP vs. CF-MSFP, F-MSFP vs. CF-MSFP, F-MSFP vs. C-MSFP) are presented in Table A1. Error bars are hidden when not visible, ±SD, *n* ≥ 3.

Investigations into the drug delivery and activity of CF-MSFPs in breast cancer cell lines yielded intriguing findings. The breast cancer cell lines used in this study represented different subtypes: MDA-MB-231 was classified as triple-negative claudin-low, MCF-7 as Luminal A, and SK-BR-3 as HER2. These various subtypes pose unique challenges in breast cancer treatment, especially in the case of triple-negative breast cancer (TNBC), which lacks hormone receptors and operates in a challenging, “cold” environment where traditional therapies struggle to be effective. Initial assessment of SFPs and MSFPs revealed low toxicity across all cell lines (Figure 7). This indicated their potential biocompatibility and safety as drug carriers. However, when we treated these cells with drugs encapsulated in MSFPs, specifically CUR (CUR) and 5-Fluorouracil (5-FU), we observed higher toxicity to cancer cells. These drugs are known for their selectivity towards cancer cells while sparing normal cells to some extent. The pronounced toxicity of the free drugs on breast cancer cells after just 24 h of treatment indicates their rapid and uncontrolled release, leading to an immediate impact on the cancer cells. The results showed that more than 70% of breast cancer cells were effectively eliminated, underscoring the potent cytotoxic effects of the free drugs within this short timeframe. This aligns with the global quest for more precise and effective breast cancer treatments, especially for challenging subtypes such as TNBC. These subtypes exhibit unique characteristics, which require innovative solutions. Nanoparticles, such as CF-MSFPs, are promising carriers for tailored breast cancer therapy. They offer the advantage of precisely delivering treatments to the site where they are needed, overcoming the obstacles presented by challenging breast cancer subtypes. By encapsulating and gradually releasing treatments, nanoparticles have the potential to carry chemotherapy drugs, immunotherapies, or hormone-like substances, ultimately improving outcomes for patients facing difficult breast cancer subtypes.

Notably, the combination of CUR and 5-FU within CF-MSFPs exhibited a synergistic effect, resulting in elevated cytotoxic responses against breast cancer cell lines (Figure 7A–F). This suggests a potential interaction between CUR and 5-FU, enhancing their individual cytotoxic effects and leading to an increased rate of cell death. The controlled drug release mechanism of CF-MSFPs may play a crucial role in providing more focused and controlled delivery of drugs, minimising off-target effects, and reducing excessive toxicity to healthy cells.

Furthermore, the impact on non-cancerous cells, represented by the HEK-293 cell line, demonstrated relatively low toxicity, with observed cell viability remaining high even at the highest tested concentrations (exceeding 200 µg/mL). This observation highlights the potential selective targeting capabilities of both the free drugs and CF-MSFPs. For all breast cancer cell lines, the concentrations required to achieve significant cytotoxic effects were around 100–150 µg/mL, except for free 5-FU in MCF-7 cells. Interestingly, MCF-7 cells exhibited a slightly higher sensitivity to free 5-FU compared to encapsulated 5-FU, indicating that MCF-7 cells may not respond as effectively to 5-FU treatment as other breast cancer cell lines. This difference in sensitivity could be attributed to the unique molecular characteristics and signalling pathways of MCF-7 cells, which may influence their response to 5-FU. Some studies have suggested that MCF-7 cells may possess enhanced DNA repair mechanisms, leading to reduced sensitivity to DNA-damaging agents, such as 5-FU [55,56]. Additionally, MCF-7 cells have a slower cell cycle and a higher percentage of cells in the G1 phase [57], which is the least susceptible phase to the cytotoxic effects of 5-FU, as this drug mainly targets cells in the S phase. Overall, these findings underscore the potential of CF-MSFPs as a promising drug delivery system for breast cancer treatment, with the potential to enhance therapeutic efficacy and minimise off-target effects in future clinical applications.

### 3.11. Cell Cycle Analysis

**Figure 8:**
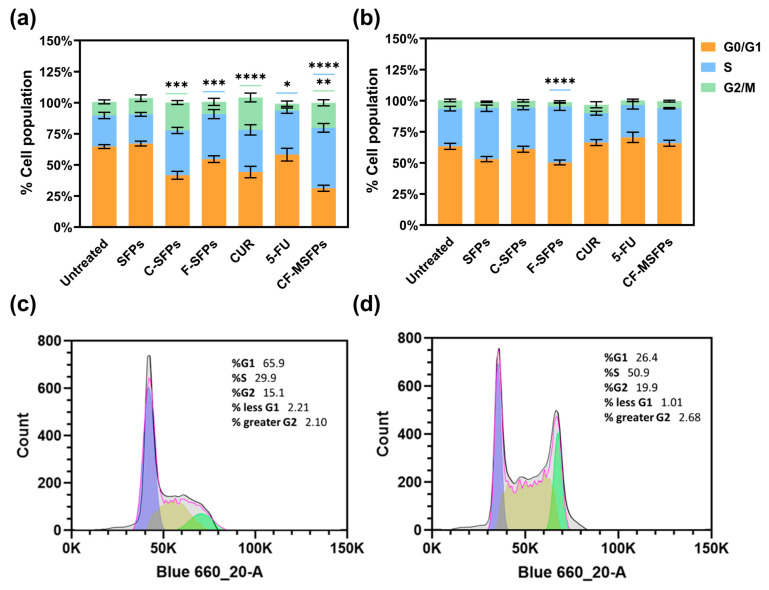
Cell cycle analysis of cancerous and non -cancerous cells treated with different MSFPs formulation and free drugs. The percentage of cell populations in different phases of the cell cycle (G1, S, G2/M) after treatment with various formulations. Particle concentration used is equal to the assumed dosage of free drug of 100 µg/mL. Statistically significant changes compared to the untreated control using one-way ANOVA with post hoc Tukey’s test for multiple comparisons indicated by asterisks, with the number of asterisks reflecting the level of significance: * *p* < 0.05, ** *p* < 0.01, *** *p* < 0.001, **** *p* < 0.0001. The colours correspond to the cell cycle phases with significant changes observed: blue for S, and green for G2/M. (**a**) MDA-MB-231 cells. (**b**) HEK-293 cells. (**c**) Representative histogram for MDA-MB-231—untreated. (**d**) Representative histogram for MDA-MB-231—CF-MSFPs. Error bars are hidden when not visible, ±SD, *n* ≥ 3.

Cell cycle analysis conducted using two different cell lines, cancerous MDA-MB-231 and non-cancerous HEK293 cells, provided interesting findings. Treatment with free CUR, C-MSFPs, and CF-MSFPs induced notable cell arrest in the G2/M phase of MDA-MB-231 cells (Figure 8). This significant effect suggests that CUR-based treatments have the potential to disrupt the cell cycle progression of cancerous cells, leading to cell cycle arrest at the G2/M checkpoint [58]. CUR can modulate various signalling pathways involved in cell cycle regulation, leading to cell cycle arrest and subsequent apoptosis in cancer cells [59]. The observed G2/M arrest is consistent with previous research findings that indicate the ability of CUR to interfere with mitotic processes and induce DNA damage, ultimately resulting in cell cycle arrest and cytotoxicity.

In contrast, treatment with 5-FU, F-MSFPs, and CD-MSFPs resulted in a significant increase in cell arrest in the S phase for MDA-MB-231 cells. This finding suggests that 5-FU-based treatments primarily affect the S phase of the cell cycle in cancer cells. The 5-FU is a chemotherapeutic agent that interferes with DNA formation by inhibiting thymidylate synthase, an enzyme essential for DNA replication [60]. As a result, cancer cells exposed to 5-FU experience S-phase arrest and DNA damage, leading to cytotoxicity and cell death. In addition to assessing cell cycle arrest, dead cells were also collected and analysed. However, the analysis did not specifically include the sub-G1 population. The sub-G1 peak typically indicates apoptotic cells with fragmented DNA, but in this study, dead cells were considered in the overall analysis without a separate focus on sub-G1. This approach allowed for a comprehensive understanding of the cell cycle dynamics and the extent of cell death induced by the treatments, particularly highlighting the cytotoxic effects of CUR and 5-FU-based treatments on MDA-MB-231 cells.

Interestingly, in non-cancerous HEK293 cells, cell cycle analysis did not show significant effects for most of the treatments, except for F-MSFPs. This discrepancy could be attributed to the differential sensitivity of cancerous and non-cancerous cells to treatments. Cancer cells often have altered cell cycle regulation and higher proliferation rates than normal cells, making them more susceptible to the effects of anticancer agents. Non-cancerous cells, such as HEK293 cells, may have intact cell cycle control mechanisms and lower sensitivity to CUR and 5-FU treatments, explaining the lack of significant changes in the cell cycle profile. Overall, the observed effects of CUR and 5-FU on cell cycle arrest align with their known mechanisms of action. Curcumin disrupts mitosis and induces DNA damage, leading to G2/M phase arrest, whereas 5-FU interferes with DNA formation, leading to S phase arrest in cancerous cells.

### 3.12. In Vivo Tolerability

**Figure 9:**
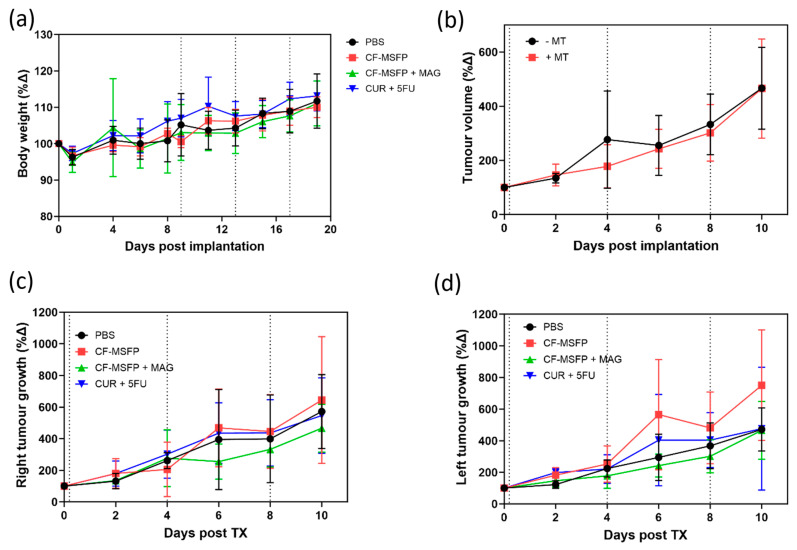
Multifaceted evaluation of treatment effects in BALB/c mice. (**a**) depicts the relative changes in body weight over 20 days post-treatment, comparing the control group with groups treated with CF-MSFPs, with and without the application of a magnetic field on the right tumour, and with the free drugs CUR and 5-FU. (**b**) shows the total tumour volume progression over 10 days in mice treated with and without magnetic targeting. (**c**,**d**) display the tumour growth dynamics over the same period for the right and left tumours, respectively, illustrating the variation in tumour response to the different treatment formulations. Significance test was conducted using one-way ANOVA with post hoc Tukey’s test but no significant difference was found. Error bars are hidden when not visible.

In this study, we investigated the tolerability of CF-MSFP, a novel drug delivery system based on magnetic targeting, in female BALB/c mice implanted with murine breast cancer cells (4T1 cells) in the mammary fat pad. The doses of free drugs used in this study were 25 mg/kg for CUR and 15 mg/kg for 5-FU based on prior efficacy studies. The doses of CF-MSFP were chosen to encapsulate the same number of free drugs in encapsulated form, assuming consistent encapsulation efficiency. However, to ensure more precise dosing, future studies should measure the in vivo drug release and adjust the administered dose accordingly. This adjustment would help achieve consistent therapeutic effects across treated groups. Then, we closely monitored the body weight of BALB/c mice after treatment with CF-MSFP. We observed that mice treated with CF-MSFP did not show a significant reduction in body weight compared to the control group. This finding presented in Figure 9 suggests that CF-MSFP treatment did not induce substantial systemic toxicity or adverse effects that would lead to significant weight loss, indicating a good safety profile for this drug delivery system. Moreover, a key advantage of CF-MSFP over conventional chemotherapy is the reduced occurrence of adverse effects. Traditional chemotherapy is known to cause various side effects owing to its non-specific nature, affecting both healthy and cancerous cells [61]. However, in our study, we observed that the use of CF-MSFP did not induce typical side effects often associated with new chemotherapy treatments such as weight loss, reduced activity, fur loss, and signs of gastrointestinal distress. This outcome indicates that the magnetic targeting strategy of CF-MSFP may offer more targeted and localised drug delivery, minimising off-target effects and reducing overall toxicity.

While this was not a survival study, the potential benefits of using magnetic guidance with CF-MSFP in reducing tumour growth were compared in both the left and right tumours. While the observed differences in tumour growth between CF-MSFP with magnetic guidance and CF-MSFP without guidance were not statistically significant, we noticed a trend of reduced tumour growth in the group treated with magnetic guidance. This trend suggests that the magnetic guidance technique may contribute to enhanced drug delivery and localised treatment at the tumour site, potentially leading to more effective tumour growth inhibition over time. Although an efficacy study was not conducted in this project, there is a trend showing that drug accumulation helped reduce tumour growth. The lack of significant differences might result from intrinsic variability in tumour response, insufficient sample size, or suboptimal magnetic guidance. To prove this hypothesis, a comprehensive efficacy study would need to be conducted. This study should include long-term monitoring of tumour growth and survival rates in animal models treated with CF-MSFPs with and without magnetic guidance. Additionally, detailed analyses involving tumour size measurements, histopathological examination, and molecular assessments to quantify drug accumulation, distribution, and therapeutic effects at various time points would be essential to validate the enhanced efficacy of the magnetic guidance technique.

### 3.13. Cell Necrosis Percentage Determination

Necrosis is a form of cell death that occurs as a result of pathological factors and plays a significant role in tumour regression and response to therapy. The quantification of necrotic tissue in the tumour sections provides valuable information about the efficacy of the treatment in inducing cell death and tissue damage within the tumour [62]. These data help evaluate the impact of treatment on tumour progression and its potential as a therapeutic strategy. The integration of magnetic nanoparticles into drug delivery represents a remarkable leap forward in the pursuit of targeted cancer therapies. When we consider the potential impact of magnetic fields in guiding these particles, it becomes evident that such an approach could significantly refine the delivery and concentration of therapeutic agents directly at the tumour site. This precision not only holds the promise of heightening the treatment’s efficacy by inducing higher rates of necrosis within the tumour tissues but also serves to curtail the collateral damage typically associated with systemic treatments.

**Figure 10:**
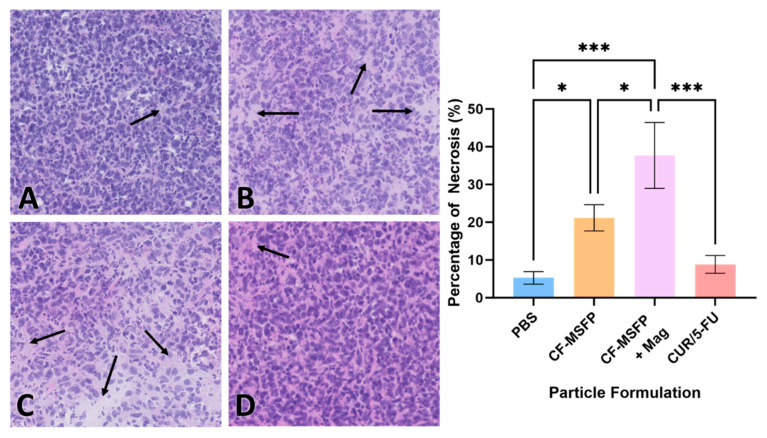
H&E staining revealed different effects on the necrosis percentage of mouse model BALB/c cells treated with various formulations. Representative images depict tissue sections from mice treated with PBS (**A**), CF-MSFP (**B**), CF-MSFP + Mag (**C**), and CUR/5-FU (**D**). The arrows in subfigures (**A**–**D**) indicate areas of necrosis within the tissue sections. corresponding graph illustrates the percentage of necrosis for all four formulations, providing a quantitative assessment of their effects on tissue integrity. Necrosis was scored by defining specific areas within tissue sections and highlighting the necrotic regions. The percentage of necrosis was calculated as the area of necrotic tissue divided by the total tissue area within the defined region, multiplied by 100. ‘*’ and ‘***’ annotations, denoting statistical significance with *p* < 0.05 and *p* < 0.001, respectively.

The necrosis visualized in the H&E staining of the BALB/c mouse model presented in Figure 10 suggests a potent effect on tumour cells when magnetic fields are employed. The CF-MSFP combined with the magnetic field demonstrates a remarkable enhancement in tissue necrosis. This aligns with the hypothesis that magnetic navigation can overcome biological barriers, allowing for a more concentrated and sustained release of the chemotherapeutic agents at the tumour site. Conversely, the comparative lack of necrosis in the tissues treated with CUR/5-FU without magnetic assistance points to the limitations of drug distribution via the bloodstream. This traditional approach, devoid of targeting or encapsulation, often leads to subtherapeutic dosing at the tumour site due to dilution effects, non-specific interactions throughout the body, or immune clearance. Hence, the future of oncological treatments could be vastly improved by such targeted delivery systems, offering hope for increased treatment specificity, reduced side effects, and improved patient outcomes.

### 3.14. CF-MSFPs Biodistribution Study

The in vivo biodistribution analysis of 5-FU and CUR, delivered via CF-MSFPs, provides valuable insights into the behaviour of these drugs, and where the drugs have concentrated in organs after CF-MSFP administration.

**Figure 11:**
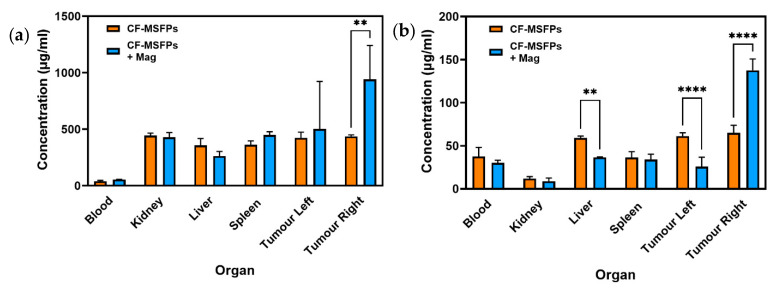
Biodistribution of CF-MSFPs in the presence and absence of an external magnetic field. Biodistribution profile of CF-MSFPs in various organs with and without an external magnetic field (Mag). (**a**) The concentration of 5-FU in micrograms per milliliter (µg/mL) across blood, kidney, liver, spleen, and left and right tumour sites. (**b**) The concentration of CUR across the same tissue sites. Increased concentration of drugs in tumour sites under the influence of a magnetic field is indicated by the ‘**’ and ‘****’ annotations, denoting statistical significance with *p* < 0.01 and *p* < 0.0001, respectively. The significance test used is one-way ANOVA with Tukey’s test for multiple comparisons.

For 5-FU, a significant increase in its concentration was observed in the right tumour, which is the site with magnetic guidance (Figure 11). This finding suggests that the magnetic targeting of CF-MSFPs effectively delivers and accumulates 5-FU specifically in the right tumour, potentially enhancing its therapeutic effects at this localised site. This magnetic-guided drug delivery approach shows promise for achieving targeted drug delivery, thereby maximising the therapeutic impact while minimising drug exposure to healthy tissues.

In contrast, the biodistribution of CUR showed much more intriguing results. Significant reductions in CUR concentration were observed in the liver and left tumour, indicating controlled and targeted delivery to these organs. This controlled release of CUR could potentially mitigate off-target effects and reduce toxicity. Remarkably, a significant increase in CUR concentration was observed in the right tumour, suggesting effective targeting and accumulation of CUR in the tumour site with magnetic guidance. The preferential accumulation of CUR in the right tumour demonstrates the potential of CF-MSFPs in achieving site-specific drug delivery, which may lead to improved therapeutic outcomes for breast cancer treatment.

The observed 2-fold increase in drug concentration within the tumour sites under the influence of an external magnetic field, while statistically significant, may not necessarily translate to a biologically significant effect in all contexts. Biological significance often depends on the specific therapeutic index of the drug and the required concentration to achieve a therapeutic effect without causing toxicity. Further studies are required to determine if this increased concentration correlates with improved therapeutic outcomes, such as enhanced tumour regression or reduced metastasis, which would establish the biological relevance of this finding. Nevertheless, the in vivo biodistribution results of CF-MSFPs loaded with 5-FU and CUR emphasise the importance of precision and targeted drug delivery in cancer therapy. The magnetic guidance of CF-MSFPs offers a promising strategy for achieving site-specific drug delivery, with preferential accumulation in the tumour site, while sparing healthy tissues. These findings highlight the potential of CF-MSFPs as an efficient drug delivery system for breast cancer treatment, warranting further investigations to optimise the magnetic targeting approach and to explore the full therapeutic potential of this nanocarrier system in preclinical and clinical settings.

## 4. Conclusions

In conclusion, our study demonstrates the effectiveness of microfluidics-assisted desolvation using a novel swirl mixer, for the fabrication of SFPs. A key aspect of this approach is its alignment with sustainability and environmental responsibility. Using silk fibroin, derived from *Bombyx mori*, as a biodegradable and non-antigenic bio-inspired material, and adopting microfluidics, especially the swirl mixer, reduces material waste and energy consumption. This technology provides precise control over mixing parameters, enabling efficient production with minimal resource usage.

The incorporation of magnetic nanoparticles and anticancer agents, such as curcumin and 5-fluorouracil, into SFPs using the swirl mixer resulted in improved encapsulation efficiency, highlighting the potential for targeted drug delivery. In vitro studies demonstrated superior cellular uptake and sustained cytotoxic effects in breast cancer cell lines, with biocompatibility observed in non-cancerous HEK293 cells. In vivo experiments further validated CF-MSFPs as a promising drug delivery platform. Magnetic guidance enhanced drug accumulation at tumour sites, achieving a two-fold increase in drug concentration and demonstrating the potential for therapeutic impact, despite the study’s design limitations in assessing long-term tumour reduction.

However, it is important to acknowledge the limitations of this microfluidics-based approach. The high initial cost of microfluidic devices compared to traditional methods may limit widespread adoption, particularly in resource-constrained settings. Additionally, scaling up production for large volumes remains challenging, especially when handling highly viscous silk fibroin solutions, which may require further optimisation of operational parameters. Maintaining nanoparticle size uniformity and encapsulation efficiency over extended production times also requires technical expertise and precise control.

Overall, the fabrication and characterisation of CF-MSFPs underscore the significance of microfluidics-assisted desolvation in developing efficient, precise, and sustainable nanoparticle systems. These findings advance the potential of CF-MSFPs in targeted drug delivery for breast cancer treatment and highlight the broader implications of combining microfluidics with bio-inspired materials. Further optimisation and extended preclinical studies are warranted to fully realise the therapeutic potential of this approach.

## Data Availability

The data presented in this study are available on request from the corresponding author.

## Appendix A

**Table A1:** Statistical analysis of key comparisons of treatments for in vitro cytotoxicity study.

Treatment Comparison	Concentration (µg/mL)	MDA-MB-231 24 h	MDA-MB-231 48 h	MCF-7 24 h	MCF-7 48 h	SK-BR-3 24 h	SK-BR-3 48 h	HEK-293 24 h	HEK-293 48 h
MSFP vs. CF-MSFP	25	****	****	ns	****	****	****	ns	**
	50	****	****	****	****	****	****	ns	****
	100	****	****	****	****	****	****	****	****
	150	****	****	****	****	****	****	****	****
	200	****	****	****	****	****	****	****	****
C-MSFP vs. CF-MSFP	25	ns	**	ns	ns	***	****	ns	ns
	50	****	****	ns	****	****	****	ns	ns
	100	ns	****	*	****	****	****	***	****
	150	***	****	****	ns	****	****	****	***
	200	**	****	****	****	****	****	****	****
F-MSFP vs. CF-MSFP	25	****	***	ns	**	****	****	ns	ns
	50	***	****	****	ns	****	****	ns	ns
	100	ns	****	****	***	****	****	ns	****
	150	ns	****	****	****	****	****	ns	ns
	200	****	****	****	****	****	****	****	****
F-MSFP vs. C-MSFP	25	**	ns	***	**	ns	****	ns	ns
	50	ns	*	****	****	ns	*	ns	ns
	100	****	***	****	****	****	****	**	ns
	150	****	***	****	****	ns	****	****	ns
	200	****	ns	****	****	**	ns	ns	ns

Significance levels are indicated as follows: ns *p* > 0.05, * *p* < 0.05, ** *p* < 0.01, *** *p* < 0.001, **** *p* < 0.0001.

## References

[1] Datta L.P., Manchineella S., Govindaraju T. (2020). Biomolecules-derived biomaterials. Biomaterials.

[2] Ganewatta M.S., Wang Z., Tang C. (2021). Chemical syntheses of bioinspired and biomimetic polymers toward biobased materials. Nat. Rev. Chem..

[3] Zhao Z., Li Y., Xie M. (2015). Silk Fibroin-Based Nanoparticles for Drug Delivery. Int. J. Mol. Sci..

[4] Solomun J.I., Totten J.D., Wongpinyochit T., Florence A.J., Seib F.P. (2020). Manual Versus Microfluidic-Assisted Nanoparticle Manufacture: Impact of Silk Fibroin Stock on Nanoparticle Characteristics. ACS Biomater. Sci. Eng..

[5] Matthew S.A.L., Rezwan R., Perrie Y., Seib F.P. (2022). Volumetric Scalability of Microfluidic and Semi-Batch Silk Nanoprecipitation Methods. Molecules.

[6] Wang S.-L., Lin S.-Y., Li M.-J., Wei Y.-S., Hsieh T.-F. (2005). Temperature effect on the structural stability, similarity, and reversibility of human serum albumin in different states. Biophys. Chem..

[7] Ruzza P., Honisch C., Hussain R., Siligardi G. (2021). Free radicals and ros induce protein denaturation by uv photostability assay. Int. J. Mol. Sci..

[8] Mattu C., Li R., Ciardelli G. (2013). Chitosan nanoparticles as therapeutic protein nanocarriers: The effect of ph on particle formation and encapsulation efficiency. Polym. Compos..

[9] Cao Z., Chen X., Yao J., Huang L., Shao Z. (2007). The preparation of regenerated silk fibroin microspheres. Soft Matter.

[10] Shi P., Goh J.C. (2011). Release and cellular acceptance of multiple drugs loaded silk fibroin particles. Int. J. Pharm..

[11] Zhang Y.-Q., Shen W.-D., Xiang R.-L., Zhuge L.-J., Gao W.-J., Wang W.-B. (2007). Formation of silk fibroin nanoparticles in water-miscible organic solvent and their characterization. J. Nanopart. Res..

[12] Lammel A.S., Hu X., Park S.-H., Kaplan D.L., Scheibel T.R. (2010). Controlling silk fibroin particle features for drug delivery. Biomaterials.

[13] Song W., Muthana M., Mukherjee J., Falconer R.J., Biggs C.A., Zhao X. (2017). Magnetic-Silk Core-Shell Nanoparticles as Potential Carriers for Targeted Delivery of Curcumin into Human Breast Cancer Cells. ACS Biomater. Sci. Eng..

[14] Zhao Z., Li Y., Chen A.-Z., Zheng Z.-J., Hu J.-Y., Li J.-S., Li G. (2013). Generation of Silk Fibroin Nanoparticles via Solution-Enhanced Dispersion by Supercritical CO_2_. Ind. Eng. Chem. Res..

[15] Leisk G.G., Lo T.J., Yucel T., Lu Q., Kaplan D.L. (2010). Electrogelation for Protein Adhesives. Adv. Mater..

[16] Ward K., Fan Z.H. (2015). Mixing in microfluidic devices and enhancement methods. J. Micromech. Microeng..

[17] Wongpinyochit T., Totten J.D., Johnston B.F., Seib F.P. (2019). Microfluidic-assisted silk nanoparticle tuning. Nanoscale Adv..

[18] Bhardwaj A., Kaur J., Wuest M., Wuest F. (2017). In situ click chemistry generation of cyclooxygenase-2 inhibitors. Nat. Commun..

[19] Li W., Chen Q., Baby T., Jin S., Liu Y., Yang G., Zhao C.-X. (2021). Insight into drug encapsulation in polymeric nanoparticles using microfluidic nanoprecipitation. Chem. Eng. Sci..

[20] Saad W.S., Prud’homme R.K. (2016). Principles of nanoparticle formation by flash nanoprecipitation. Nano Today.

[21] Zhu D., Long Q., Xu Y., Xing J. (2019). Evaluating nanoparticles in preclinical research using microfluidic systems. Micromachines.

[22] Tomeh M.A., Mansor M.H., Hadianamrei R., Sun W., Zhao X. (2022). Optimization of large-scale manufacturing of biopolymeric and lipid nanoparticles using microfluidic swirl mixers. Int. J. Pharm..

[23] Gao Z., Mansor M.H., Winder N., Demiral S., Maclnnes J., Zhao X., Muthana M. (2023). Microfluidic-Assisted ZIF-Silk-Polydopamine Nanoparticles as Promising Drug Carriers for Breast Cancer Therapy. Pharmaceutics.

[24] Ayub Z.H., Arai M., Hirabayashi K. (1993). Mechanism of the Gelation of Fibroin Solution. Biosci. Biotechnol. Biochem..

[25] Hu X., Kaplan D., Cebe P. (2006). Determining Beta-Sheet Crystallinity in Fibrous Proteins by Thermal Analysis and Infrared Spectroscopy. Macromolecules.

[26] Besenhard M.O., LaGrow A.P., Hodzic A., Kriechbaum M., Panariello L., Bais G., Loizou K., Damilos S., Margarida Cruz M., Thanh N.T.K. (2020). Co-precipitation synthesis of stable iron oxide nanoparticles with NaOH: New insights and continuous production via flow chemistry. Chem. Eng. J..

[27] Perteghella S., Crivelli B., Catenacci L., Sorrenti M., Bruni G., Necchi V., Vigani B., Sorlini M., Torre M.L., Chlapanidas T. (2017). Stem cell-extracellular vesicles as drug delivery systems: New frontiers for silk/curcumin nanoparticles. Int. J. Pharm..

[28] Seib F.P., Jones G.T., Rnjak-Kovacina J., Lin Y., Kaplan D.L. (2013). pH-Dependent Anticancer Drug Release from Silk Nanoparticles. Adv. Healthc. Mater..

[29] Sharma S., Bano S., Ghosh A.S., Mandal M., Kim H.-W., Dey T., Kundu S.C. (2016). Silk fibroin nanoparticles support in vitro sustained antibiotic release and osteogenesis on titanium surface. Nanomed. Nanotechnol. Biol. Med..

[30] Shaidani S., Jacobus C., Sahoo J.K., Harrington K., Johnson H., Foster O., Cui S., Hasturk O., Falcucci T., Chen Y. (2023). Silk Nanoparticle Synthesis: Tuning Size, Dispersity, and Surface Chemistry for Drug Delivery. ACS Appl. Nano Mater..

[31] Hasturk O., Sahoo J.K., Kaplan D.L. (2020). Synthesis and characterization of silk ionomers for layer-by-layer electrostatic deposition on individual mammalian cells. Biomacromolecules.

[32] Wongpinyochit T., Uhlmann P., Urquhart A.J., Seib F.P. (2015). PEGylated Silk Nanoparticles for Anticancer Drug Delivery. Biomacromolecules.

[33] Kundu J., Chung Y.-I., Kim Y.H., Tae G., Kundu S.C. (2010). Silk fibroin nanoparticles for cellular uptake and control release. Int. J. Pharm..

[34] Wongpinyochit T., Johnston B.F., Seib F.P. (2016). Manufacture and Drug Delivery Applications of Silk Nanoparticles. J. Vis. Exp..

[35] Asapur P., Mahapatra S.K., Banerjee I. (2022). Secondary structural analysis of non-mulberry silk fibroin nanoparticles synthesized by using microwave and acetone method. J. Biomol. Struct. Dyn..

[36] Zhao Z., Chen A., Li Y., Hu J., Liu X., Li J., Zhang Y., Li G., Zheng Z. (2012). Fabrication of silk fibroin nanoparticles for controlled drug delivery. J. Nanopart. Res..

[37] Hu Y., Zhang Q., You R., Wang L., Li M. (2012). The Relationship between Secondary Structure and Biodegradation Behavior of Silk Fibroin Scaffolds. Adv. Mater. Sci. Eng..

[38] Alarcon R., Walter M., Paez M., Azócar M.I. (2023). Ostwald Ripening and Antibacterial Activity of Silver Nanoparticles Capped by Anti-Inflammatory Ligands. Nanomaterials.

[39] Matthew S.A., Totten J.D., Phuagkhaopong S., Egan G., Witte K., Perrie Y., Seib F.P. (2020). Silk nanoparticle manufacture in semi-batch format. ACS Biomater. Sci. Eng..

[40] Tavares M.R., de Menezes L.R., do Nascimento D.F., Souza D.H.S., Reynaud F., Marques M.F.V., Tavares M.I.B. (2016). Polymeric nanoparticles assembled with microfluidics for drug delivery across the blood-brain barrier. Eur. Phys. J. Spec. Top..

[41] Balas F., Manzano M., Horcajada P., Vallet-Regí M. (2006). Confinement and controlled release of bisphosphonates on ordered mesoporous silica-based materials. J. Am. Chem. Soc..

[42] Shi L., Zhang J., Zhao M., Tang S., Cheng X., Zhang W., Li W., Liu X., Peng H., Wang Q. (2021). Effects of polyethylene glycol on the surface of nanoparticles for targeted drug delivery. Nanoscale.

[43] Hans M.L., Lowman A.M. (2002). Biodegradable nanoparticles for drug delivery and targeting. Curr. Opin. Solid. State Mater. Sci..

[44] Luo R., Neu B., Venkatraman S.S. (2012). Surface functionalization of nanoparticles to control cell interactions and drug release. Small.

[45] Wang Y., Li P., Kong L. (2013). Chitosan-modified PLGA nanoparticles with versatile surface for improved drug delivery. AAPS PharmSciTech.

[46] Manuja A., Kumar B., Athira S., Sarkar P., Riyesh T., Kumar N., Tripathi B.N., Mann B. (2022). Zinc oxide nanoparticles encapsulated in polysaccharides alginate/gum acacia and iron oxide nanomatrices show enhanced biocompatibility and permeability to intestinal barrier. Food Hydrocoll. Health.

[47] Del Bianco L., Spizzo F., Yang Y., Greco G., Gatto M.L., Barucca G., Pugno N.M., Motta A. (2022). Silk fibroin films with embedded magnetic nanoparticles: Evaluation of the magneto-mechanical stimulation effect on osteogenic differentiation of stem cells. Nanoscale.

[48] Greco G., Schmuck B., Del Bianco L., Spizzo F., Fambri L., Pugno N.M., Veintemillas-Verdaguer S., Morales M.P., Rising A. (2024). High-performance magnetic artificial silk fibers produced by a scalable and eco-friendly production method. Adv. Compos. Hybrid. Mater..

[49] Li H., Zhu J., Chen S., Jia L., Ma Y. (2017). Fabrication of aqueous-based dual drug loaded silk fibroin electrospun nanofibers embedded with curcumin-loaded RSF nanospheres for drugs controlled release. RSC Adv..

[50] Li H., Tian J., Wu A., Ge C., Sun Z. (2016). Self-assembled silk fibroin nanoparticles loaded with binary drugs in the treatment of breast carcinoma. Int. J. Nanomed..

[51] Yu L. (2001). Amorphous pharmaceutical solids: Preparation, characterization and stabilization. Adv. Drug Deliv. Rev..

[52] Craig D.Q. (2002). The mechanisms of drug release from solid dispersions in water-soluble polymers. Int. J. Pharm..

[53] Howard F., Al-Janabi H., Patel P., Cox K., Smith E., Vadakekolathu J., Pockley A.G., Conner J., Nohl J.F., Allwood D.A. (2022). Nanobugs as Drugs: Bacterial Derived Nanomagnets Enhance Tumor Targeting and Oncolytic Activity of HSV-1 Virus. Small.

[54] Ni W., Li Z., Liu Z., Ji Y., Wu L., Sun S., Jian X., Gao X. (2019). Dual-Targeting Nanoparticles: Codelivery of Curcumin and 5-Fluorouracil for Synergistic Treatment of Hepatocarcinoma. J. Pharm. Sci..

[55] Fujimori A., Gupta M., Hoki Y., Pommier Y. (1996). Acquired camptothecin resistance of human breast cancer MCF-7/C4 cells with normal topoisomerase I and elevated DNA repair. Mol. Pharmacol..

[56] Watanabe T., Oba T., Tanimoto K., Shibata T., Kamijo S., Ito K.-I. (2021). Tamoxifen resistance alters sensitivity to 5-fluorouracil in a subset of estrogen receptor-positive breast cancer. PLoS ONE.

[57] Osborne C.K., Hobbs K., Trent J.M. (1987). Biological differences among MCF-7 human breast cancer cell lines from different laboratories. Breast Cancer Res. Treat..

[58] Liu E., Wu J., Cao W., Zhang J., Liu W., Jiang X., Zhang X. (2007). Curcumin induces G2/M cell cycle arrest in a p53-dependent manner and upregulates ING4 expression in human glioma. J. Neuro-Oncol..

[59] Zheng M., Ekmekcioglu S., Walch E.T., Tang C.-H., Grimm E.A. (2004). Inhibition of nuclear factor-κB and nitric oxide by curcumin induces G2/M cell cycle arrest and apoptosis in human melanoma cells. Melanoma Res..

[60] Li M.-H., Ito D., Sanada M., Odani T., Hatori M., Iwase M., Nagumo M. (2004). Effect of 5-fluorouracil on G1 phase cell cycle regulation in oral cancer cell lines. Oral Oncol..

[61] Liu Y.-Q., Wang X.-L., He D.-H., Cheng Y.-X. (2021). Protection against chemotherapy-and radiotherapy-induced side effects: A review based on the mechanisms and therapeutic opportunities of phytochemicals. Phytomedicine.

[62] Ormerod M.G., Sun X.-M., Brown D., Snowden R.T., Cohen G.M. (1993). Quantification of apoptosis and necrosis by flow cytometry. Acta Oncol..

